# Aminothioneine, a product derived from golden oyster mushrooms (*Pleurotus cornucopiae* var. *citrinopileatus*), activates Ca^2+^ signal-mediated brain-derived neurotrophic factor expression in cultured cortical neurons

**DOI:** 10.1016/j.bbrep.2021.101185

**Published:** 2021-12-13

**Authors:** Mamoru Fukuchi, Kazuki Watanabe, Satoru Mitazaki, Momoko Fukuda, Satoshi Matsumoto

**Affiliations:** a Laboratory of Molecular Neuroscience, Faculty of Pharmacy, Takasaki University of Health and Welfare, 60 Nakaorui-machi, Takasaki, Gunma, 370-0033, Japan; b Laboratory of Natural Medicines, Faculty of Pharmacy, Takasaki University of Health and Welfare, 60 Nakaorui-machi, Takasaki, Gunma, 370-0033, Japan; c LS Corporation Co., Ltd, 13-4 Nihonbashi Kodenma-cho, Chuo-ku, Tokyo, 103-0001, Japan

**Keywords:** BDNF, Ca^2+^ signal, CREB, CRTC1, Golden oyster mushroom, NMDAR

## Abstract

Ameliorating reduced brain-derived neurotrophic factor (BDNF) expression or maintaining high BDNF levels in the brain has been suggested to improve brain function in neurological diseases and prevent aging-related brain dysfunction. In this study, we found that a food-derived product, Aminothioneine® (AT), which is prepared from the extract of golden oyster mushrooms (*Pleurotus cornucopiae* var. *citrinopileatus*), increased *Bdnf* mRNA expression levels in primary rat cortical neuron cultures. Ergothioneine (ET) comprises more than 1% in AT and is an active constituent of AT, and ET has been reported to increase neurotrophin-4/5, but not BDNF, expression levels in neural stem cells. ET also did not affect *Bdnf* mRNA expression in cultured cortical neurons, suggesting that AT contains other active constituents that induce *Bdnf* mRNA expression in neurons. AT-induced *Bdnf* mRNA expression was completely blocked by *d*-(−)-2-Amino-5-phosphonopentanoic acid but partially blocked by nicardipine, U0126, and FK506. This result suggested that *N*-methyl-*d*-aspartate receptor-derived Ca^2+^ signals, including those mediated by extracellular signal-regulated kinase/mitogen-activated protein kinase and calcineurin, are the main contributors to *Bdnf* mRNA induction. In addition, AT increased cAMP-response element-binding protein (CREB) phosphorylation and the nuclear localization of CREB-regulated transcriptional coactivator 1 in neurons. Thus, AT can increase *Bdnf* mRNA expression via Ca^2+^ signal-induced CREB-dependent transcription in neurons. Because AT is a food-derived product, increasing and/or maintaining BDNF levels in the brain by daily intake of the product could be possible, which may be beneficial for neurological and aging-related disorders.

## Highlights

• Aminothioneine® (AT) induced *Bdnf* mRNA expression in cultured rat cortical neurons.
• Ergothioneine tended to induce *Nt-4/5* but did not affect *Bdnf* mRNA expression.
• AT activated MAPK and calcineurin-regulated CREB-dependent transcription.

## Abbreviations

APV: *d*-(−)-2-Amino-5-phosphonopentanoic acid
AT: Aminothioneine®
BDNF: brain-derived neurotrophic factor
CaMK: Ca^2+^/calmodulin-dependent protein kinase
CREB: cAMP-response element-binding protein
CRTC1: CREB-regulated transcriptional coactivator 1
ERK: extracellular signal-regulated kinase
ET: ergothioneine
GOME: concentrated extracts of golden oyster mushrooms
MAPK: mitogen-activated protein kinase
NGF: nerve growth factor
NMDAR: *N*-methyl-*d*-aspartate receptor
NMR: nuclear magnetic resonance
NT-3: neurotrophin-3
NT-4/5: neurotrophin-4/5

## 1 Introduction

Previous reports obtained from postmortem brains, clinical studies, and animal experiments strongly suggested that reduced brain-derived neurotrophic factor (BDNF) expression levels in the brain are associated with neurodegenerative diseases and neuropsychiatric disorders [1]. BDNF levels have been reported to be decreased in the brains of patients with major depressive disorder, bipolar disorder, schizophrenia, Alzheimer’s disease, and Parkinson’s disease [2]. Because BDNF plays a crucial role in a variety of neural functions such as synaptic plasticity [3], it is plausible that reduced BDNF levels in the brain cause neural dysfunctions resulting in these neurological diseases. Therefore, BDNF may be a drug target for these diseases. For example, hexadecanamide has been reported to upregulate hippocampal BDNF expression and improve cognitive functions in a mouse model of Alzheimer’s disease [4]. 7,8-Dihydroxyflavone, which is an agonist of tropomyosin-related kinase B (TrkB), a BDNF and neurotrphin-4/5 (NT-4/5) receptor, also has similar beneficial effects on Alzheimer’s disease model mice [5]. In addition, the hippocampal BDNF levels in patients using antidepressant medications were higher than those in patients not using these medications [6]. Thus, maintaining a certain BDNF level in the brain could suppress the onset and progression of neurological disorders.

On the basis of these findings, we aimed to identify substances that induce BDNF expression in neuronal cells. For this purpose, we investigated the effects of natural products, extracts prepared from plants and herbal medicines, and chemical compounds on the expression of *Bdnf* mRNA using primary cultures of cortical neurons. Recently, we found Aminothioneine® (AT), a product derived from golden oyster mushrooms (*Pleurotus cornucopiae* var. *citrinopileatus*), induced *Bdnf* mRNA expression in neurons. AT contains more than 1% of ergothioneine (ET), which is a food-derived hydrophilic amino acid and an antioxidant [7], and ET is an active constituent of AT. Previously, ET and the extract prepared from golden oyster mushrooms were demonstrated to exhibit antidepressant-like effects in mice [8]. In addition, ET has beneficial effects on cognitive function not only in mice [9] but also in healthy individuals [10]. One mechanism underlying the beneficial effects of ET could be that ET induces NT-4/5 expression and subsequently activates TrkB signaling in neural stem cells [11], resulting in enhancement of neuronal differentiation [8]. Although the effects of ET on neural stem cells have been investigated, the effects of AT and ET on neurons remain unclear. In this study, we examined the effects of AT and ET on primary cultured neurons by focusing on the expression of neurotrophins. We report that AT, but not ET, significantly increased *Bdnf* mRNA expression by activating *N*-methyl-*d*-aspartate receptor (NMDAR)-derived Ca^2+^ signals, which can activate cAMP-response element-binding protein (CREB)-dependent transcription in neurons. Thus, AT can induce BDNF expression in neurons, independent of the effect of ET. These findings suggest that AT could exhibit beneficial effects, such as its antidepressant-like effect and cognitive improvement, by inducing BDNF expression in neurons, in addition to ET-mediated neuronal differentiation in neural stem cells.

## 2 Materials and methods

### 2.1 Materials

AT was kindly donated by LS Corporation Co., Ltd. (Tokyo, Japan). Briefly, AT was prepared by extraction from golden oyster mushrooms (*Pleurotus cornucopiae* var. *citrinopileatus*) in hot water, and then the extract was concentrated. The concentrated extract was mixed with dextrin, and the mixture was freeze-dried (AT is the powder obtained after freeze-drying). Because AT contains dextrin as an additive, we also used concentrated extracts of golden oyster mushrooms (GOME) that did not contain dextrin as an additive. ET, dextrin (from maize starch, catalog No. 31410), *d*-(−)-2-Amino-5-phosphonopentanoic acid (*d*-APV), FK506, KN93, nicardipine, and U0126 were purchased from Sigma-Aldrich (St. Louis, MO, USA).

### 2.2 Fractionation

GOME (20.8 g) was evaporated *in vacuo*, the residue (5.6 g, yield from GOME: 27.0%) was fractionated by Diaion HP-20 (Mitsubishi-chemical, Tokyo, Japan) column and then successively eluted with MeOH–H_2_O (3:7, v/v), MeOH, and EtOAc (each solvent volume 2 L) to obtain a 30% MeOH eluate fraction (GOME-1) (4.27 g, 20.5%), MeOH eluate fraction (GOME-2) (728 mg, 3.5%), and EtOAc eluate fraction (GOME-3) (5.1 mg, 0.025%), respectively. GOME-2 fraction was fractionated by preparative HPLC using H_2_O as the mobile phase at a flow rate of 1.5 mL/min to give six fractions [GOME-2-1 (150 mg, 0.72%), GOME-2-2 (81.5 mg, 0.39%), GOME-2-3 (43.7 mg, 0.21%), GOME-2-4 (85.9 mg, 0.41%), GOME-2-5 (1.5 mg, 0.0072%), and GOME-2-6 (204 mg, 0.98%)]. HPLC was performed using a system composed of a L-6000 pump (Hitachi, Tokyo, Japan), a Shodex RI-71 (Showa-Denko, Tokyo, Japan) detector, and a Rheodyne injection port. A Capcell Pak C_18_ AQ column (10 × 250 mm, 3 μm particle size) (Shiseido, Tokyo, Japan) was used for the preparative HPLC.

### 2.3 Cell culture

Primary cultures of rat cortical neurons were prepared from cerebral cortices of Sprague-Dawley rats at embryonic day 17 [Japan SLC (Shizuoka, Japan)], as described previously [12,13]. All animal care and experimental protocols were approved by the Animal Experiment Committee of Takasaki University of Health and Welfare (Authorization No. 1733, 1809, 1913, and 2008), and were performed in accordance with the Guidelines for the Care and Use of Laboratory Animals of Takasaki University of Health and Welfare. The cells were seeded (1.8 × 10^6^ cells) and cultured in poly-*l*-lysine-coated 6-well plates [AGC Techno Glass (Shizuoka, Japan)] for RT-PCR, and 8 × 10^5^ cells were cultured on 18-mm-diameter poly-*l*-lysine-coated coverslips [Matsunami Glass Ind., Ltd., (Osaka, Japan)] in 12-well plates (AGC Techno Glass) for immunostaining. The cells were cultured using Neurobasal medium [Thermo Fisher Scientific (Waltham, MA, USA)] containing B27 supplement (Thermo Fisher Scientific), 2 μg/mL gentamicin (Thermo Fisher Scientific), and 0.5 mM glutamine (Thermo Fisher Scientific). Half of the culture medium was replaced with fresh medium every 3 days. Each experiment was performed at 13 days in culture.

### 2.4 RT-PCR

**Fig. 1:**
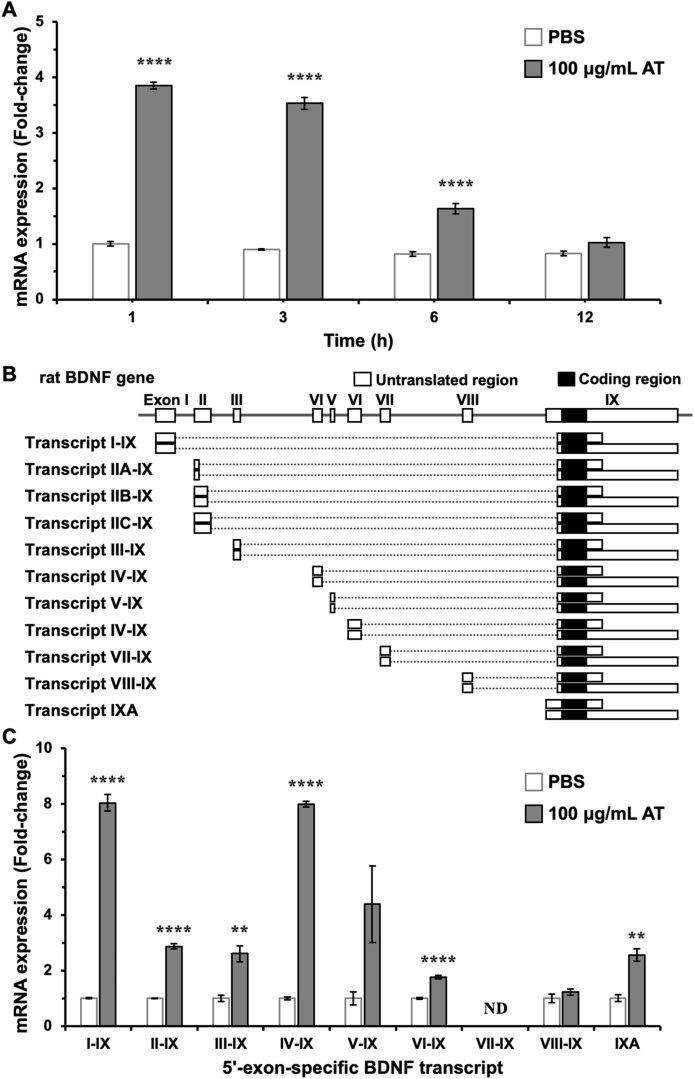
Aminothioneine (AT)-induced *Bdnf* mRNA expression in cultured neurons. (A) Cultured rat cortical neurons at 13 days *in vitro* were treated with 100 μg/mL AT or PBS (a solvent of AT), and then total RNA was isolated at the indicated time points (1, 3, 6, and 12 h after the treatment). Changes in *Bdnf* mRNA expression were examined by RT-PCR. ****p < 0.0001 versus PBS at the same time points (n = 3, two-way ANOVA with Sidak’s multiple comparisons test). (B) Structure of rat *Bdnf* gene and its multiple transcripts. (C) Total RNA was isolated 3 h after the treatment of cultured neurons with 100 μg/mL AT or PBS, and changes in 5’ exon-specific *Bdnf* mRNA expression were examined by RT-PCR. **p < 0.01 and ****p < 0.0001 versus PBS (n = 3, unpaired *t*-test). ND; not detected.

One microgram of total RNA, which was isolated and purified using an ISOSPIN Cell & Tissue RNA kit [Nippongene (Tokyo, Japan)], was reverse-transcribed into cDNA using a PrimeScript 1st strand cDNA Synthesis Kit [Takara Bio Inc. (Shiga, Japan)], according to the manufactures’ instructions. Real-time PCR was carried out using SYBR Select Master Mix (Thermo Fisher Scientific), as described previously [12,13]. PCR thermal profiles included initial heating at 50 °C for 2 min then at 95 °C for 2 min, followed by 45 cycles of denaturation at 95 °C for 45 s, annealing at 57 °C for 45 s, and extension at 72 °C for 1 min. Fold-change values were calculated by the ^ΔΔ^Ct method to determine relative gene expression. The Primer sequences were as follows: rat *Gapdh*-forward: 5’-ATCGTGGAAGGGCTCATGAC-3’, rat *Gapdh*-reverse: 5’-TAGCCCAGGATGCCCTTTAGT-3’, rat total *Bdnf*-forward: 5’-CTGGAGAAAGTCCCGGTATCAA-3’, rat total *Bdnf*-reverse: 5’-TTATGAACCGCCAGCCAATTCTCTT-3’, rat *Ngf*-forward: 5’-GCTGAACCAATAGCTGCCC-3’, rat *Ngf*-reverse: 5’-GAAGTCTAAATCCAGAGTGTCCG-3’, rat *Nt-3*-forward: 5’-CCAAGCAGATGGTAGATGTTAAGG-3’, rat *Nt-3*-reverse: 5’-GCCGTAGTAGTTCTGTGTCTG-3’, rat *Nt-4/5*-forward: 5’-CAGTGTGCGATGCAGTGAG-3’, rat *Nt-4/5*-reverse: 5’-CGCGTCTCGAAGAAGTACTG-3’. A 5’ exon-specific forward primer and exon IX common reverse primer [12] were used to detect each exon-specific *Bdnf* transcript (Fig. 1B and C).

### 2.5 Immunostaining

The cells were fixed in PBS containing 4% formaldehyde and 4% sucrose for 15 min at room temperature and then treated with blocking PBS containing 3% bovine serum albumin and 3% normal goat serum for 1 h at room temperature. After blocking, the cells were incubated with primary antibodies against MAP2 [Sigma-Aldrich, 1:1000 (M4403)] and CREB phosphorylated at serine 133rd [Cell Signaling Technology, Inc. (Danvers, MA, USA), 1:1000 (9198)], or CRTC1 antiserum [kindly donated by professor Hiroshi Takemori (Graduate School of Engineering, Gifu University, Gifu, Japan), 1:1000]. After washing, the cells were incubated with a CF488A- and CF568-conjugated secondary antibody against rabbit or mouse IgG, respectively [Biotium, Inc. (Fremont, CA, USA), 1:1000]. Nuclei were counter-stained with 500 nM 4’,6-diamidino-2-phenylindole (Thermo Fisher Scientific). After another wash, coverslips were mounted on slides using Fluoromount [Diagnostic BioSystems (Pleasanton, CA, USA)]. Confocal fluorescent images were obtained with a Nikon A1 confocal microscope [Nikon (Tokyo, Japan)]. The number of MAP2- and phospho-CREB-positive neurons or MAP2- and nuclear localized CRTC1-positive neurons were counted as described previously [14], and the percentages of phospho-CREB- and nuclear localized CRTC1-positive neurons were calculated.

### 2.6 Statistical analysis

All data are presented as the mean ± the standard error of the mean (S.E.M.). Statistical analyses were performed using Prism 7 software [GraphPad (San Diego, CA, USA)]. Detailed information is shown in the figure legends.

## 3 Results

### 3.1 AT increases *Bdnf* mRNA expression in cultured cortical neurons

A previous study showed that AT and its active constituent, ET, promoted neuronal differentiation and exerted antidepressant-like effects in mice [8]. Because ET has been reported to increase NT-4/5 expression in neural stem cells [11], we predicted that AT would control the expression of neurotrophic factors in neurons. Here, we focused on the BDNF expression, which fundamentally contributes to controlling numerous brain functions [3]. *Bdnf* mRNA expression was significantly increased 1 and 3 h after the treatment of primary cultured rat cortical neurons with AT (Fig. 1A). Among multiple *Bdnf* transcripts (Fig. 1B) [15], a marked induction of exon I- and exon IV-containing *Bdnf* mRNA was observed (Fig. 1C). The expression of these mRNAs is controlled by *Bdnf* promoter I and promoter IV, respectively. These promoters are well-known as neuronal activity-regulated promoters [16], which suggests that AT could activate neuronal activity-regulated transcription pathways.

**Fig. 2:**
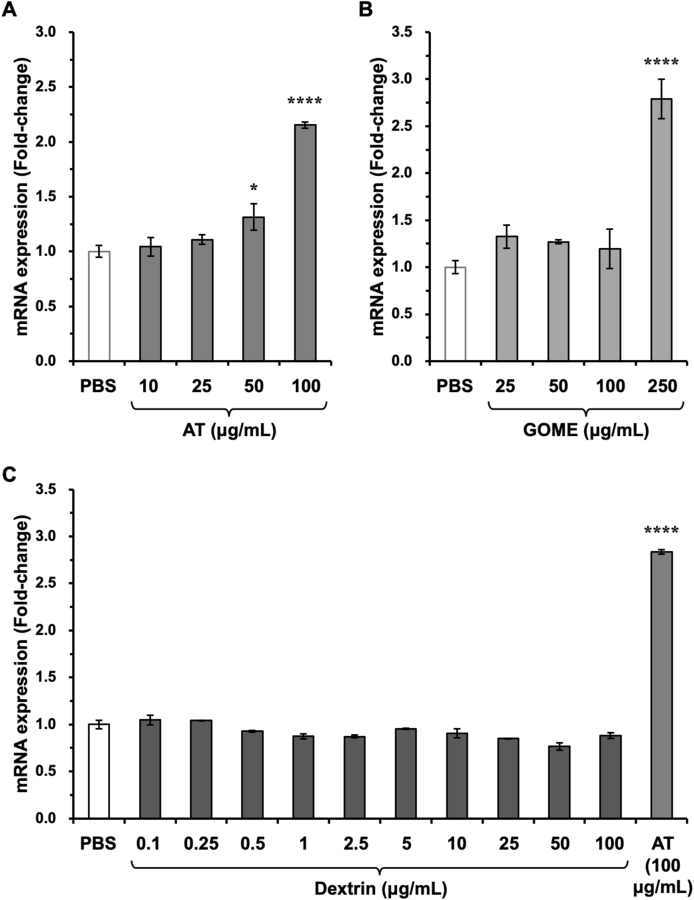
The concentrated extracts of golden oyster mushroom (GOME) also induced *Bdnf* mRNA expression in neurons. At 13 days *in vitro*, cultured rat cortical neurons were treated with AT (A), GOME (B), or dextrin (C), at the indicated final concentrations for 3 h, and total RNA was extracted to examine the changes in *Bdnf* mRNA expression by RT-PCR analysis. In Fig. 2C, AT (100 μg/mL) was used as a positive control. *p < 0.05 and ****p < 0.0001 versus PBS (n = 3, one-way ANOVA with Dunnett’s multiple comparisons test).

AT consists of a powder obtained by concentrating hot water extracts of golden oyster mushroom, adding dextrin, and then freeze-drying. To exclude the possibility of the contribution of dextrin to the AT-induced *Bdnf* mRNA expression, we also examined the effects of concentrated extracts of golden oyster mushroom (GOME) that did not contain dextrin on *Bdnf* mRNA expression in cultured neurons. Both AT and GOME increased *Bdnf* mRNA expression in a dose-dependent manner (Fig. 2A and B). In addition, we confirmed that dextrin did not increase *Bdnf* mRNA expression in cultured neurons (Fig. 2C). *Bdnf* mRNA expression was induced by AT at a final concentration of 100 μg/mL, whereas it was induced by GOME at a final concentration of 250 μg/mL. GOME used in this study is a liquid concentrate, which is prepared from golden oyster mushrooms. We obtained 5.6 g of the residue after the evaporation of 20.8 g of GOME *in vacuo* (yield from GOME: 27.0%) (see Materials and methods), suggesting that GOME would contain more than 70% moisture. Because AT is produced by freeze-drying GOME, the active ingredients would be more concentrated. This may explain why a lower concentration of AT could induced *Bdnf* mRNA expression.

### 3.2 ET could not induce *Bdnf* mRNA expression in cultured cortical neurons

**Fig. 3:**
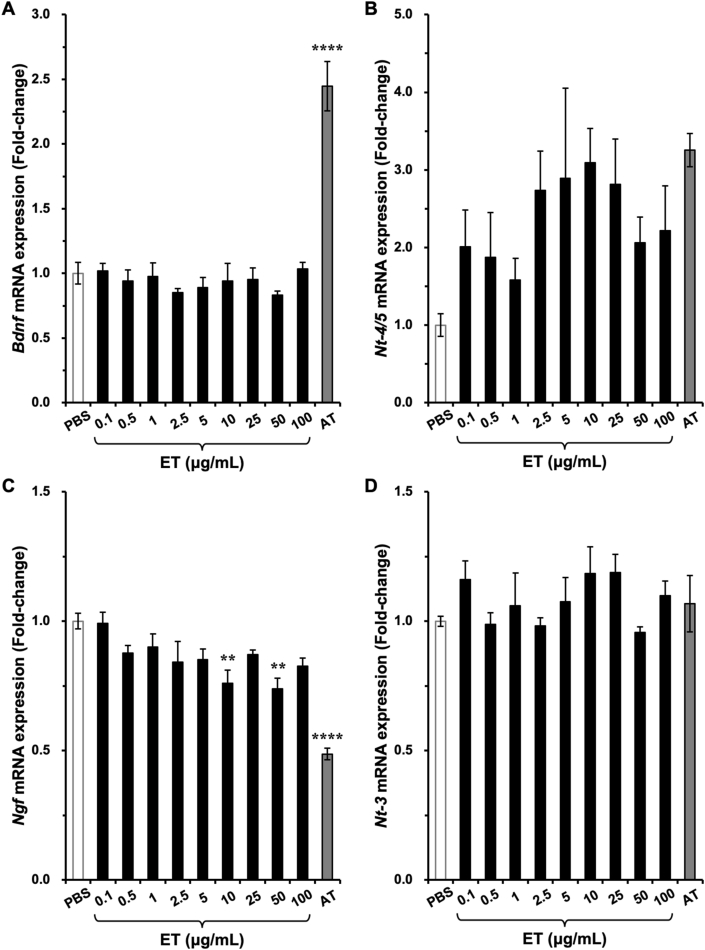
Effects of ergothioneine (ET) on neurotrophin expression in neurons. At 13 days *in vitro*, cultured rat cortical neurons were treated with ET at the indicated final concentrations or 100 μg/mL AT for 3 h. Total RNA was extracted to examine the changes in *Bdnf* (A), *Nt-4/5* (B), *Ngf* (C), and *Nt-3* (D) mRNA expression by RT-PCR. **p < 0.01 and ****p < 0.0001 versus PBS (n = 3, one-way ANOVA with Dunnett’s multiple comparisons test).

Previously, ET, which is a major active constituent of AT, was reported to increase NT-4/5 expression in neural stem cells [11]. Thus, we next examined whether the expression of *Bdnf* and other neurotrophins was altered when cultured cortical neurons were treated with ET. Because ET comprises more than 1% in AT, the final concentration of ET was determined in the range of 0.1–100 μg/mL. Although *Bdnf* mRNA expression was significantly induced by AT, no induction of *Bdnf* mRNA was observed when neurons were treated with ET (Fig. 3A). *Nt-4/*5 mRNA expression tended to be increased by ET and AT; however, no significant induction was observed (Fig. 3B). ET tended to decrease nerve growth factor (*Ngf*) mRNA expression in a dose-dependent manner, and *Ngf* mRNA was significantly decreased by AT (Fig. 3C). We did not observe changes in neurotrophin-3 (*Nt-3*) mRNA expression in the presence of ET or AT (Fig. 3D). Among these neurotrophins, *Nt-4/*5 mRNA expression was lower than that of other mRNAs [average of Ct values (PBS-treated neurons): *Gapdh* = 17.2, *Ngf* = 32.1, *Bdnf* = 26.2, *Nt-3* = 30.1, *Nt-4/5* = 39.4].

### 3.3 AT activates *Bdnf* mRNA expression via an NMDAR-CREB-dependent pathway

**Fig. 4:**
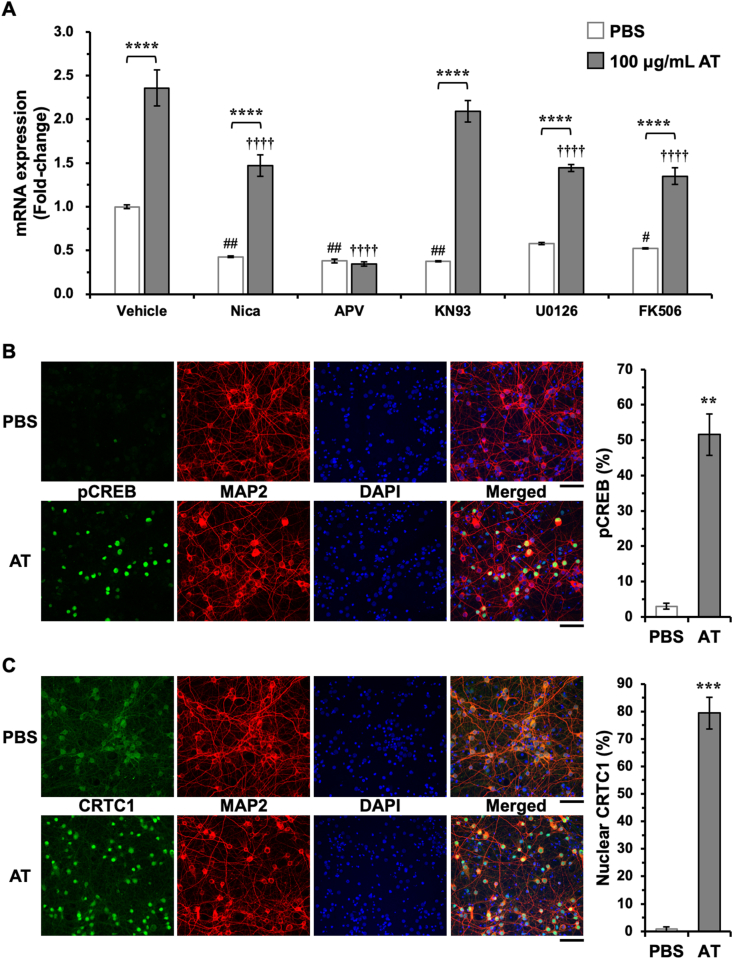
Signaling pathways involved in AT-induced *Bdnf* mRNA expression in neurons. (A) Ten minutes before treatment with 100 μg/mL AT or PBS, nicardipine (Nica, 5 μM), APV (200 μM), KN93 (10 μM), U0126 (20 μM), or FK506 (5 μM) was added to the neurons. Total RNA was prepared 3 h after AT treatment, and the changes in *Bdnf* mRNA were examined by RT-PCR. ****p < 0.0001 versus PBS, #*p* < 0.05 and ##*p* < 0.01 versus vehicle/PBS, and ††††*p* < 0.0001 versus vehicle/AT (n = 3, two-way ANOVA with Tukey’s multiple comparisons test). (B) At 13 days *in vitro*, cultured rat cortical neurons were treated with 100 μg/mL AT for 30 min, and the changes in CREB phosphorylation at Ser133 and CRTC1 localization in neurons were examined by immunostaining. Scale bar = 50 μm **p < 0.01 and ***p < 0.001 versus PBS (n = 3, unpaired *t*-test).

We next investigated intracellular signaling pathways that contribute to AT-induced *Bdnf* mRNA expression in neurons. Because a marked induction of exon I- and exon IV-containing *Bdnf* mRNA, both of which are controlled by neuronal activity-regulated promoters [16], was observed (Fig. 1B), we focused on activity-induced Ca^2+^ signaling pathways. AT-induced *Bdnf* mRNA expression was completely prevented by the NMDAR antagonist APV and partially prevented by L-type voltage-dependent Ca^2+^ channel blocker nicardipine (Fig. 4A), indicating a major contribution of NMDAR to induction of *Bdnf* mRNA expression. Among Ca^2+^ signaling pathways in cultured cortical neurons, we previously reported that Ca^2+^/calmodulin-dependent protein kinase (CaMK), extracellular signal-regulated kinase (ERK)/mitogen-activated protein kinase (MAPK), and calcineurin were mainly involved in the activation of NMDAR-dependent *Bdnf* mRNA expression [17]. Here, we found that AT-induced *Bdnf* mRNA expression was partially inhibited by U0126 (a MAPK/ERK kinase 1/2 inhibitor) and FK506 (a calcineurin inhibitor), but not by KN93 (a CaMK inhibitor), suggesting that its induction was regulated by ERK/MAPK and calcineurin.

ERK/MAPK and calcineurin activate CREB-dependent transcription [18]. ERK1/2 is an upstream kinase of ribosomal S6 kinase and mitogen- and stress-activated protein kinase, both of which are CREB kinases and therefore phosphorylate CREB at Ser133. CREB phosphorylation activates transcription of its target genes. CREB-dependent transcription is also controlled by CREB-regulated transcriptional coactivator 1 (CRTC1). Phosphorylated CRTC1 is captured by 14-3-3 protein and is mainly localized in the cytoplasm. When phosphorylated CRTC1 is de-phosphorylated by the Ca^2+^-dependent protein phosphatase calcineurin, CRTC1 translocates from the cytoplasm to the nucleus and subsequently activates CREB-dependent transcription in a phosphorylated CREB-independent manner [[19], [20], [21]]. Because BDNF is a CREB target gene [14,17,22,23], we examined the changes in CREB phosphorylation at Ser133 and CRTC1 localization in cultured cortical neurons treated with AT using immunostaining. We found few phosphorylated CREB-positive cells when the neurons were treated with PBS (Fig. 4B). In contrast, phosphorylated CREB-positive cells were significantly increased by AT treatment (Fig. 4B). In PBS-treated neurons, CRTC1 expression was diffusely detected in the cytoplasm; however, CRTC1 was mainly localized in the nucleus after AT treatment (Fig. 4C). Increases in phosphorylated CREB and nuclear localized CRTC1 were observed in MAP2-positive cells (Fig. 4B and C), indicating that AT activated CREB-dependent transcription in neurons.

## 4 Discussion

In this study, we found that AT, the product of concentrated extracts of golden oyster mushrooms, increased *Bdnf* mRNA expression in cultured cortical neurons. This increase was dependent on NMDAR, ERK/MAPK, and calcineurin, and AT treatment increased CREB phosphorylation and nuclear localization of CRTC1 in neurons. These results strongly suggest that AT activates NMDAR-derived ERK/MAPK and calcineurin pathways to induce *Bdnf* mRNA expression, which is also mediated by CREB-dependent transcription, in neurons.

Although AT contains dextrin as an additive, we also found that GOME, which does not contain dextrin, significantly increased *Bdnf* mRNA levels in cultured cortical neurons. This result suggests that the dextrin added to AT does not participate in the induction of *Bdnf* mRNA expression in neurons. In support, dextrin did not affect *Bdnf* mRNA expression. A previous study showed that ET, a major constituent of AT, increased *Nt-4/*5 mRNA expression, but not that of other neurotrophins, in neural stem cells [11]. Similar to a previous report, we found that ET did not alter *Bdnf* mRNA levels in cultured cortical neurons. Although *Nt-4/*5 mRNA levels tended to be higher after ET treatment, no significant induction of *Nt-4/*5 mRNA expression was observed in neurons, which could be because of the difference of *Nt-4/*5 mRNA expression levels in neural stem cells and primary cortical neurons. *Nt-4/*5 mRNA expression was detected using RT-PCR; however, the Ct values of *Nt-4/*5 mRNA were higher than those of the other neurotrophin mRNAs, suggesting that the endogenous *Nt-4/5* expression levels are low in neurons. Therefore, the fold-change values of each sample varied, and significant induction of *Nt-4/*5 mRNA was not observed in cultured cortical neurons. Because ET increased *Nt-4/5* but not *Bdnf* mRNA levels in neural stem cells, NT-4/5 is suggested to be the main contributor to ET-induced neuronal differentiation in neural stem cells.

In this study, we could not identify the active constituents that are involved in AT-induced *Bdnf* mRNA expression in cultured cortical neurons. However, we prepared 30% methanol, methanol, and ethyl acetate elute fractions from GOME (Fig. S1A) and found that the methanol fraction (GOME-2) in particular induced *Bdnf* mRNA expression (Fig. S1B). Because both GOME and GOME-2 increased *Bdnf* mRNA expression in a concentration-dependent manner (Fig. S1B), we further focused on GOME-2 in this study. Further fractionations of GOME-2 revealed that, among six fractions, GOME-2-2 could increase *Bdnf* mRNA expression in neurons (Fig. S1C). The following were observed in the proton nuclear magnetic resonance (^1^H NMR) spectrum of ET: an *N*-methyl group signal [N(CH_3_)_3_], a methylene group signal (H-2), a methine group signal (H-3a and H-3b), and an olefin group signal (H-5) (Fig. S2). When the ^1^H NMR spectra of GOME-2, GOME-2-1, GOME-2-2, GOME-2-3, and GOME-2-4 were compared by focusing on the olefin group signal at H-5 of ET, this olefin group signal was observed in GOME-2 and GOME-2-4, but not in GOME-2-1, GOME-2-2, and GOME-2-3 (Fig. S2). Thus, the ^1^H NMR spectra analysis strongly suggested that GOME-2-2, which is the active fraction, does not contain ET. Thus, these findings revealed that ET did not contribute to AT-induced *Bdnf* mRNA expression in cultured cortical neurons. Therefore, AT may have other active constituents that induce *Bdnf* mRNA expression in neurons. Further investigations are necessary to identify these active constituents. On the other hand, GOME-2-3, GOME-2-4, GOME-2-5, and GOME-2-6 reduced *Bdnf* mRNA expression, whereas GOME-2-2 significantly induced *Bdnf* mRNA expression in neurons (Fig. S1C). Therefore, GOME-2 might contain multiple constituents which could activate and inhibit *Bdnf* transcription. Because inhibitory constituents might be concentrated in GOME-2-3, GOME-2-4, GOME-2-5, and GOME-2-6 by fractionation of GOME-2, these fractions might have reduced *Bdnf* mRNA expression. In any case, we did not further examine the effects of these fractions on the expression of *Bdnf* mRNA in neurons because they did not increase *Bdnf* mRNA expression.

Several previous studies support the idea that the transcriptional machinery involved in BDNF expression could be altered in the brains of patients with these neurological diseases. For example, amyloid β prevented the expression of CRTC1/CREB-dependent neuronal genes including *Bdnf*, which likely resulted in hippocampal-dependent memory impairment in mouse and rat models of Alzheimer’s disease [24,25]. Although wild type huntingtin protein can interact with REST/NRSF and increase BDNF expression, mutant huntingtin, which has been found in Huntington’s disease patients, loses the ability to interact with REST/NRSF, resulting in repression of *Bdnf* and other REST/NRSF target genes [26,27]. In a chronic stress-induced depression model in mice, the levels of salt-inducible kinase 2, which is a kinase of CRTC1, was increased in the hippocampus, resulting in inhibition of CRTC1/CREB-regulated BDNF expression [28]. Therefore, enhancing *Bdnf* transcription in these pathological conditions may result in therapeutic effects, and agents that can induce BDNF expression in neurons may be candidate therapeutic drugs for neurological diseases. Because AT is a food-derived product, daily intake of AT may confer resistance to stress-induced or aging-related neurological diseases such as depression and dementia, which is likely mediated by AT-induced BDNF expression in neurons in addition to ET-mediated neurogenesis in neural stem cells.

## Conflicts of interest

The authors declare no competing interests.

## Declaration of competing interest

The authors declare that they have no known competing financial interests or personal relationships that could have appeared to influence the work reported in this paper.
