# Supplementary material for: Aminothioneine, a product derived from golden oyster mushrooms (Pleurotus cornucopiae var. citrinopileatus), activates Ca2+ signal-mediated brain-derived neurotrophic factor expression in cultured cortical neurons
Source: Biochem Biophys Rep. 2021 Dec 13;28:101185. doi: 10.1016/j.bbrep.2021.101185 (PMC8683675; doi:10.1016/j.bbrep.2021.101185)
Supplement: Multimedia component 1 [file mmc1.pdf]

Supplementary Data

Aminothioneine, a product derived from golden oyster mushrooms (*Pleurotus cornucopiae* var. *citrinopileatus*), activates Ca^{2+} signal-mediated Brain-derived neurotrophic factor expression in cultured cortical neurons

Mamoru Fukuchi[a,†], Kazuki Watanabe[b], Satoru Mitazaki[a], Momoko Fukuda[a], Satoshi Matsumoto[c]

[a]Laboratory of Molecular Neuroscience, Faculty of Pharmacy, Takasaki University of Health and Welfare, 60 Nakaorui-machi, Takasaki, Gunma 370-0033, Japan

[b]Laboratory of Natural Medicines, Faculty of Pharmacy, Takasaki University of Health and Welfare, 60 Nakaorui-machi, Takasaki, Gunma 370-0033, Japan

[c]LS Corporation Co., Ltd, 13-4 Nihonbashi Kodenma-cho, Chuo-ku, Tokyo 103-0001, Japan

[†]Corresponding author: Mamoru Fukuchi

Laboratory of Molecular Neuroscience, Faculty of Pharmacy, Takasaki University of Health and Welfare, 60 Nakaorui-machi, Takasaki, Gunma 370-0033, Japan

Tel: +81-27-352-1180 (ext. 8317); Fax: +81-27-352-1118; E-mail: fukuchi@takasaki-u.ac.jp

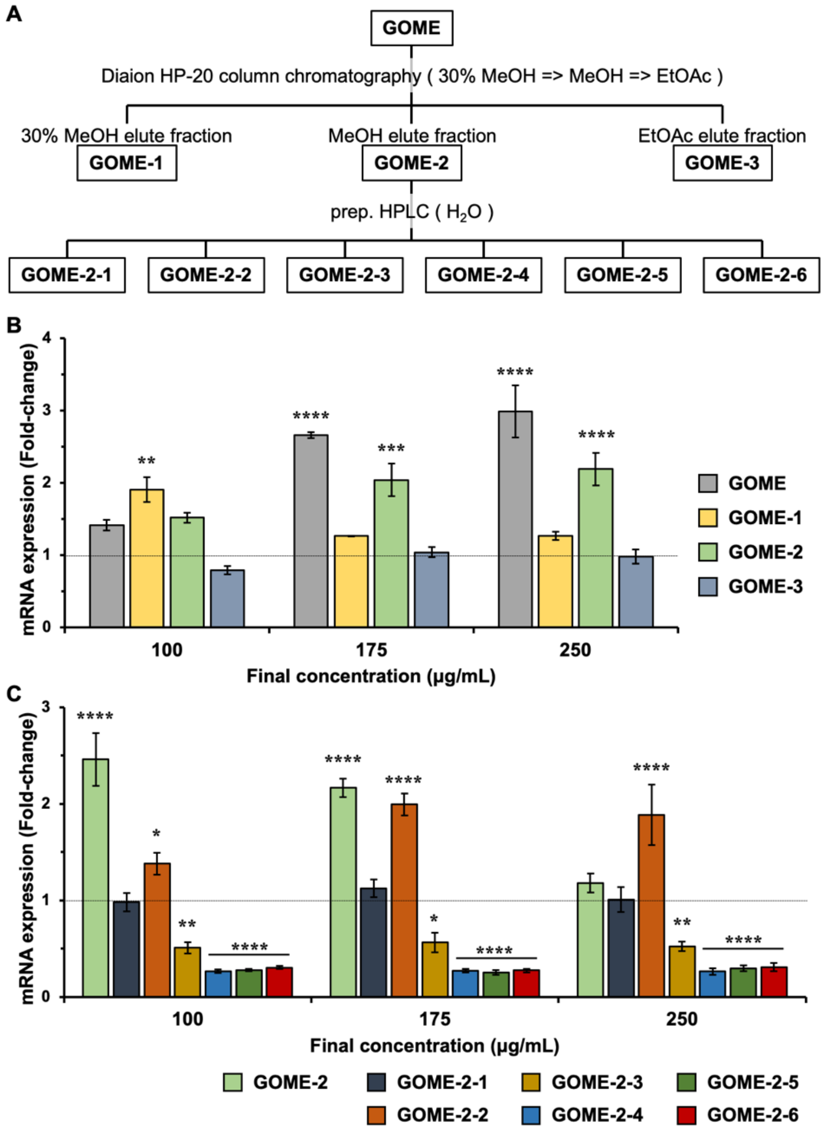

Figure S1. Effect of eluate fractions prepared from extracts of golden oyster mushrooms (GOME) on *Bdnf* mRNA expression in cultured neurons.

(A) An outline for fractionation. Detailed information was shown in Materials and methods. (B, C) At 13 days *in vitro*, cultured rat cortical neurons were treated with GOME-1, GOME-2, and GOME-3 (B), or GOME-2, GOME-2-1, GOME-2-2, GOME-2-3, GOME-2-4, GOME-2-5, and GOME-2-6 (C) at the indicated final concentrations for 3 h. Total RNA was extracted to examine the changes in *Bdnf* mRNA expression by RT-PCR. We determined the fold-change values of *Bdnf* mRNA expression by each fraction by comparing with the level of *Bdnf* mRNA expression in the PBS-treated neurons. * $p < 0.05$, ** $p < 0.01$, *** $p < 0.001$, and **** $p < 0.0001$ versus PBS (n = 3, one-way ANOVA with Dunnett's multiple comparisons test).

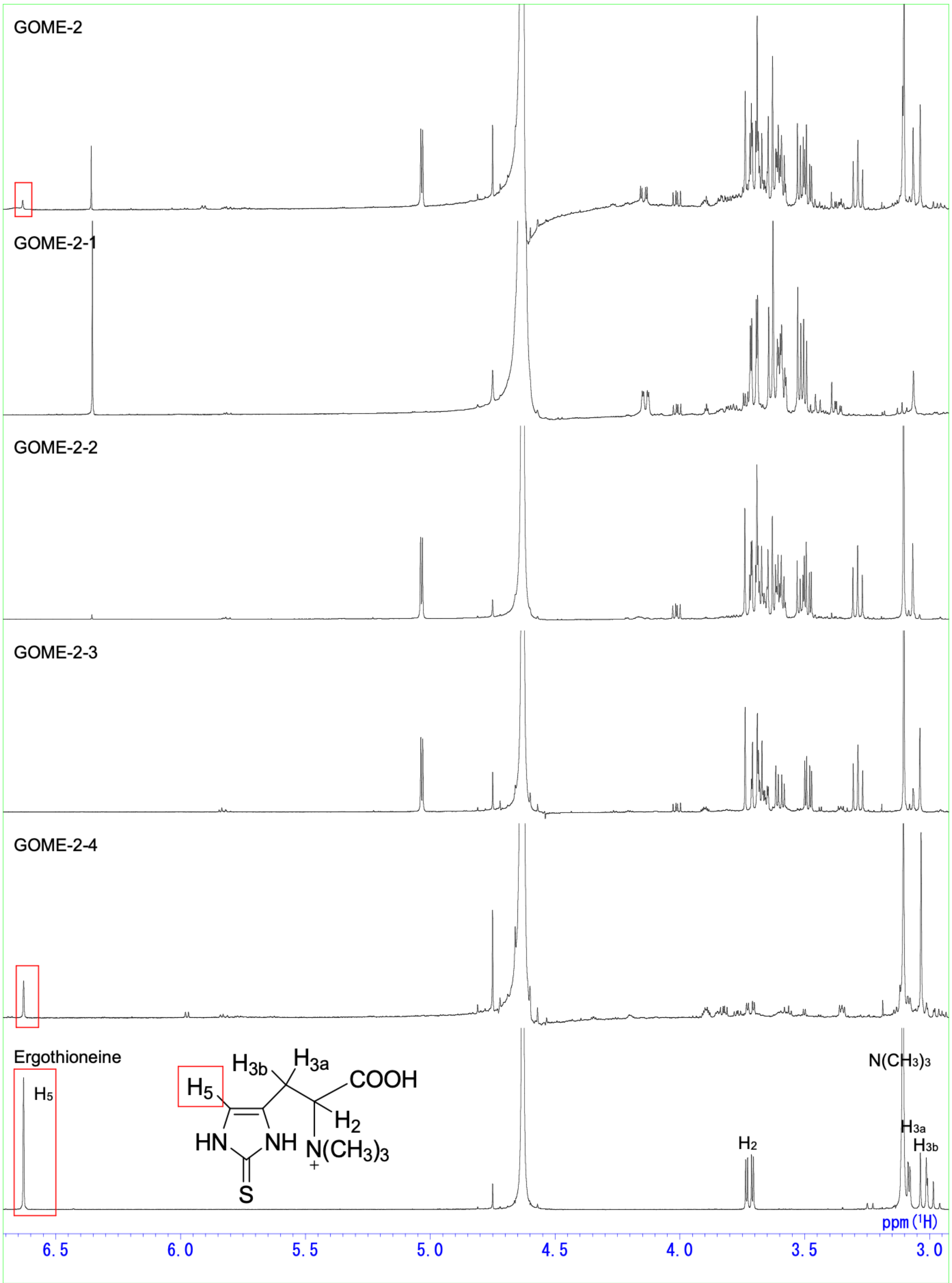

Figure S2. Proton nuclear magnetic resonance (^{1}H-NMR) spectra of GOME-2, GOME-2-1, GOME-2-2, GOME-2-3, GOME-2-4, and ergothioneine in D_2O.

NMR spectra were recorded on a JEOL ECZ-500 (500 MHz for ^{1}H NMR, Tokyo, Japan) spectrometer using standard JEOL pulse programs.
